# Risk factors for nasal malignancies in German men: the South-German Nasal cancer study

**DOI:** 10.1186/1471-2407-12-506

**Published:** 2012-11-06

**Authors:** Eberhard M Greiser, Karin Halina Greiser, Wolfgang Ahrens, Rudolf Hagen, Roland Lazszig, Heinz Maier, Bernhard Schick, Hans Peter Zenner

**Affiliations:** 1 Center for Social Policy Research, Faculty of Health Sciences, Bremen University, Bremen, Germany; 2 Epi.Consult GmbH Ortsstr. 1 A, 54534, Musweiler, Germany; 3 Bremen Institute for Prevention Research and Social Medicine (BIPS), Bremen University (until 3-2004), Bremen, Germany; 4 German Cancer Research Center, Division of Cancer Epidemiology, Heidelberg, Germany; 5 BIPS - Institute for Epidemiology and Prevention Research, Bremen, Germany; 6 Institute for Statistics, Bremen University, Bremen, Germany; 7 Department of Otorhinolaryngology, University of Wuerzburg, Wuerzburg, Germany; 8 Department of Otorhinolaryngology-Head and Neck Surgery, Albert-Ludwigs University Medical School, Freiburg, Germany; 9 Department of Otorhinolaryngology, Head and Neck Surgery, Bundeswehr Hospital, Ulm, Germany; 10 Department of Otorhinolaryngology, Saarland University, Homburg/Saar, Germany; 11 Department of Otolaryngology, University of Tübingen, Tübingen, Germany

**Keywords:** Case–control study, Nasal cancer, Smoking, Hardwood dust, Asbestos, Organic solvents, Insecticides, Nasal spray, Nasal lavage, Nasal snuff

## Abstract

**Background:**

There are few studies of the effects of nasal snuff and environmental factors on the risk of nasal cancer. This study aimed to investigate the impact of using nasal snuff and of other risk factors on the risk of nasal cancer in German men.

**Methods:**

A population-based case–control study was conducted in the German Federal States of Bavaria and Baden-Württemberg. Tumor registries and ear, nose and throat departments provided access to patients born in 1926 or later.

**Results:**

Telephone interviews were conducted with 427 cases (mean age 62.1 years) and 2.401 population-based controls (mean age 60.8 years). Ever-use of nasal snuff was associated with an odds ratio (OR) for nasal cancer of 1.45 (95% confidence interval [CI] 0.88–2.38) in the total study population, whereas OR in smokers was 2.01 (95% CI 1.00-4.02) and in never smokers was 1.10 (95% CI 0.43–2.80). The OR in ever-smokers vs. never-smokers was 1.60 (95% CI 1.24–2.07), with an OR of 1.06 (95% CI 1.05–1.07) per pack-year smoked, and the risk was significantly decreased after quitting smoking. Exposure to hardwood dust for at least 1 year resulted in an OR of 2.33 (95% CI 1.40–3.91) in the total population, which was further increased in never-smokers (OR 4.89, 95% CI 1.92–12.49) in analyses stratified by smoking status. The OR for nasal cancer after exposure to organic solvents for at least 1 year was 1.53 (1.17–2.01). Ever-use of nasal sprays/nasal lavage for at least 1 month rendered an OR of 1.59 (1.04–2.44). The OR after use of insecticides in homes was 1.48 (95% CI 1.04–2.11).

**Conclusions:**

Smoking and exposure to hardwood dust were confirmed as risk factors for nasal carcinoma. There is evidence that exposure to organic solvents, and in-house use of insecticides could represent novel risk factors. Exposure to asbestos and use of nasal snuff were risk factors in smokers only.

## Background

It is generally agreed that all kind of tobacco products are both addictive and carcinogenic. This also includes smokeless tobacco products [1,2]. There are, however, few publications investigating the association between the use of snuff and nasal cancer.

In a case series of cancers of the paranasal sinuses (CPNS) in Bantu males from Transvaal, South Africa, 22 out of 28 had used nasal snuff for a prolonged time [3]. In another case series [4] on cancers of the nasal cavity (CNC) and CPNS in the boot and shoe industry in Northamptonshire, Great Britain, eight out of 26 patients were nasal snuff users.

In three subsequent publications, results of case–control studies have been reported.

In a case–control study including 160 patients with CNC or CPNS and 290 hospital controls from four hospitals in North Carolina and Virginia, an increased hazard ratio of 1.47 (95% confidence interval [CI] 0.8–2.8) for use of snuff was detected [5]. A case–control study from the Florida Cancer Registry was based on 71 cases of nasopharyngeal cancer (NPC), on 92 cases of CPNS and CNC, and on 8.285 controls with cancers unrelated to tobacco use [6]. The reported odds ratios (OR) for use of snuff were 5.3 (95% CI 0.7–41.6) for NPC and 3.3 (95% CI 0.4–25.9) for CNC/CPNS. However, in the United States, snuff is often taken orally, so the results of the latter two case–control studies provide only marginal evidence for estimating the risks of nasal use of snuff.

A recent case–control study from North Africa (Algeria, Morocco, Tunisia) [7] reported on the impact of tobacco and cannabis use on NPC risk in 636 cases and 625 hospital/family/neighborhood controls. The OR for ever-use of snuff, either orally or nasally, for all histologic types of nasal cancer was 1.03 (95% CI 0.64–1.65), but was 30.2 (95% CI 1.67–546) for differentiated carcinomas. The latter result is obviously based on very few cases, as indicated by the extremely large CI.

We conducted a population-based case–control study to investigate the impact of nasal snuff on the risk of CNC, CPNS and NPC. Simultaneously we analyzed the potential impact of individual, environmental and occupational risk factors for this group of malignancies. The study was conducted by the Bremen Institute for Prevention Research and Social Medicine (BIPS), Bremen, Germany.

## Methods

Sales figures provided by a major manufacturer indicated that the use of nasal snuff in Germany was most prevalent in two Southern Federal States of Germany, Bavaria and Baden-Württemberg, thus these areas were chosen as the study region. The study design was approved by the Ethical Review Committees of the Bremen State Chamber of Physicians, of the Bavarian State Chamber of Physicians, of the Baden-Württemberg State Chamber of Physicians, of Augsburg Central Hospital, and of Tübingen University Hospital. Design, conduct and analysis of the study were overseen by an independent scientific advisory board (see Appendix). Cases were restricted to male patients born after 1925 with primary CNC, CPNS, or NPC. Diagnoses were confirmed histologically starting January 1, 1990, and cases were recruited retrospectively. Access to patients was achieved through clinical tumor registries in Bavaria, and through ear, nose and throat (ENT) departments in all hospitals treating such patients in Baden-Württemberg. Histopathologic findings for each case were provided by the respective tumor registry or by ENT departments. In both federal states, the ENT departments sought written consent from patients to participate in telephone interviews. In hospitals without a link to a clinical tumor registry, archives of hospital records were perused by hospital staff to identify eligible patients. Age-matched controls were drawn from community residency registries of communities with resident cases or of communities of comparable size within the same federal state. As residency registries comprise in each German community the complete resident population, the community administrators were asked for a random sample of resident males with sample characteristics specified by number for each age. It was not possible to reconstruct the status of residency registries according to the year of diagnosis of cases. Sample size was calculated for a prevalence of 0.12 to allow for detection of ORs of 1.4 with type I and type II errors of 0.05 and 0.80, respectively. This suggested 700 cases and 2100 controls with 1:3 frequency matching for year of birth +/- 2 years. During the first phase of the study it became obvious that a sample of 700 cases could not be reached, but the calculated number of controls was adhered to. The calculated OR did not change to a relevant extent, but it was assumed that the precision regarding confounders would increase.

The questionnaire consisted of sociodemographic variables, lifetime occupational history, use of nasal snuff, smoking, exposure to environmental tobacco smoke, diseases of the nose, previous malignancies, family history of malignancies, nutritional habits including consumption of alcohol, specific exposures (use of insecticides in homes, organic solvents). Lifetime occupational history included each occupational period by year, line of business, job title and specific exposures (solvents, radioactive substances, radiation, asbestos, dusts). Additional questions were added for work in forestry, farming, carpentry, and metal-working. For each of these occupations, specific exposures were assessed by duration and type of exposures, e.g., in carpentry for type of wood (softwood, hardwood, chipboard wood).

During a pilot study, face-to-face interviews were conducted in cases of the Munich Tumor Registry. Logistic considerations led to the decision to use telephone interviews for the main study. A computer-assisted telephone interview system with trained and supervised female interviewers was used. Interviews of next-of-kin of deceased cases were attempted with a modified questionnaire. During the course of the study it became obvious that some of the cases had speech impediments owing to surgical therapy. These cases (n = 36) were provided with printed questionnaires by mail. Another case could not provide a telephone number and received a printed questionnaire subsequently. After completion of the pilot study, the Scientific Advisory Board suggested a modification of the food frequency list.

Statistical analyses were performed using SAS 9.2 (SAS Institute, Cary, NC, USA). for tabulation of study characteristics (Proc Summary) and for multivariate logistic regression (Proc Phreg). Assuming a linear dose–response relationship, logistic regression analyses for continuous independent variables were conducted in three different ways: ever-exposed, quantitative exposure (pack-years for smoking [i.e., smoking one pack per day for 1 year], package-years for nasal snuff [i.e. using one package per week for 1 year]), exposure years (for hardwood dust, organic solvents, asbestos, use of nasal sprays or nasal lavage), and quartiles of exposure. Additional analyses included stratification by smoking status. Adjustment for potential confounders included: year of birth; community size; educational attainment; cigarette pack-years; nasal snuff package-years; years of exposure to hardwood dust, asbestos, or organic solvents; use of nasal spray/nasal lavage; and ever-use of insecticides in homes.

## Results

Altogether, 917 potential cases were identified. After classifying these by location and histopathologic findings, 707 patients met the inclusion criteria. Interviews could be conducted with 389 (55%) of these patients, and 38 interviews of cases conducted during the pilot phase were added to the final database. Three of these cases had to be re-interviewed for application of additional occupational exposure questionnaires. Addresses of 4.207 potential controls were drawn from residency registers in Bavaria and Baden-Württemberg. Of these addresses 165 of these were no longer valid, another 68 persons were deceased. When being contacted for interviews 99 persons declined due to physical impairment. Finally 2 of the potential controls were patients who met inclusion criteria as cases. They were included as cases, after histopathologic findings had been provided by their respective hospitals. Of 3.873 eligible controls 2.401 interviews were completed, giving a response rate of 62.0%. The final study population consisted of 427 cases (mean age 62.1 [standard deviation 11.9 years]) and of 2401 controls (mean age 60.8 years [standard deviation 12.9 years]). The major characteristics of the study population are presented in Table 1. Cases had significantly lower educational attainment than controls.

**Table 1:** Major characteristics of the study population

**Year of birth**	**Cases (%)**	**Controls (%)**
< 1930	292 (12.2)	63 (14.8)
1930–39	859 (35.8)	160 (37.5)
1940–49	577 (24.0)	96 (22.5)
1950–59	367 (15.3)	64 (15.0)
1960–69	199 (8.3)	37 (8.7)
1970+	107 (4.5)	7 (1.6)
Total	427 (100.0)	2.401 (100.0)
**Community size**		
< 5.000	134 (31.4)	728 (30.3)
5.000 < 10.000	83 (19.4)	435 (18.1)
10.000 < 100.000	127 (29.7)	739 (30.8)
100.000+	83 (19.4)	499 (20.8)
	Chi^2^ = 0.92/p=0.82	
**Educational attainment**		
Primary school or less	289 (67.7)	1329 (55.4)
Secondary school	77 (18.0)	625 (26.0)
College	61 (14.3)	447 (18.6)
	Chi^2^ = 22.8/p ≤0.0001	
**Site of malignancies**		
Nasal cavity	112	-
Paranasal sinuses	135	-
Nasopharynx	180	-

Most of the tumors originated from the nasopharynx (n = 181, 42.4%) followed by the paranasal sinuses (n = 159, 37.2%) and the nasal cavity (n = 87, 20.4%). Results by location of the respective tumors are reported in brief; detailed results by malignancy location as well as by histologic classification will be published in a separate paper.

### Nasal snuff

Table 2 presents the analyses of the use of nasal snuff and the risk of nasal cancer. There was a significantly increased risk for the 4^th^ quartile of snuff package-years (OR 2.41, 95% CI 1.13–5.15) compared with never-users of nasal snuff. Stratified analyses by smoking status showed a divergent picture. In never-smokers there was no increase in risk in any of the analyses of snuff use. In smokers, the risk increased in ever-use of snuff (OR 2.01, 95% CI 1.00–4.02), per year of snuff use (OR 1.02, 95% CI 1.01–1.04), and in the 4^th^ quartile of snuff package-years (OR 6.91, 95% CI 2.31–20.7). Regarding tumor site, the increases in risk were exclusively in tumors of the paranasal sinuses (ever-use: OR 3.19, 95% CI 1.46–6.96; ever-use in smokers: OR 8.23, 95% CI 2.23–34.91; increase per year of use in all subjects: OR 1.01, 95% CI 1.00–1.02; in smokers: OR 1.03, 95% CI 1.01–1.05).

**Table 2:** Association of nasal snuff and smoking with nasal malignancies

	**Controls (%)**	**Cases (%)**	**OR (95% CI)**
**All study participants**			
Reference^*^	2273 (94.7)	399 (93.4)	**1.00**
Nasal snuff (ever)	128 (5.3)	28 (6.6)	1.45 (0.88–2.38)
Nasal snuff package-years^§^	128 (5.3)	28 (6.6)	1.01 (1.00–1.02)
Snuff package-years (quartiles) < 2	31 (1.3)	2 (0.5)	0.52 (0.10–2.68)
2 < 4	27 (1.3)	5 (1.2)	1.16 (0.36–3.75)
4 <12.5	37 (1.1)	9 (2.1)	1.29 (0.53–3.12)
12.5+	33 (1.4)	12 (2.8)	**2.41 (1.13–5.15)**
**Smokers**			
Reference^*^	1333 (94.2)	272 (93.2)	**1.00**
Nasal snuff (ever)	82 (5.8)	20 (6.8)	**2.01 (1.00–4.02)**
Nasal snuff package-years^§^	82 (5.8)	20 (6.8)	**1.02 (1.01–1.04)**
Snuff package-years(quartiles) < 2	16 (1.1)	1 (0.3)	0.60 (0.04–8.33)
2 < 4	19 (1.3)	3 (1.0)	0.75 (0.11–4.96)
4 <12.5	26 (1.8)	7 (2.4)	1.36 (0.44–4.25)
12.5+	21 (1.5)	9 (3.1)	**6.91 (2.31–2 0.69)**
**Never-smokers**			
Reference^*^	940 (95.3)	127 (94.1)	1.00
Nasal snuff (ever)	46 (4.7)	8 (5.9)	1.10 (0.43–2.80)
Nasal snuff package-years^§^	46 (4.7)	8 (5.9)	1.00 (0.98–1.02)

* never used nasal snuff.

^§^ One package-year = use of 1 package per week for one year.

Adjustments: Year of birth, community size, educational attainment, cigarette packyears (all study participants only), exposure to hardwood dust, asbestos, organic solvents, use of nasal spray/nasal lavage and ever-use of insecticides in homes.

### Smoking

Cigarette smoking emerged as a strong risk factor for nasal cancer in all parameters analyzed (Table 3). There was a 60% increase in risk in ever-smokers (95% CI 24–107%), and an increase of 6% per pack-year (95% CI 5–7%). Similar risk increases were found for tumors in all sites (data not shown).

**Table 3:** Association of smoking with nasal malignancies

	**Controls (%)**	**Cases (%)**	**OR (95% CI)**
**All study participants**			
Reference^*^	986 (41.1)	135 (31.6)	**1.00**
Smoking (ever)	1415 (58.9)	292 (68.4)	**1.60 (1.24–2.07)**
Smoking (per pack-year)	1415 (58.9)	292 (68.4)	**1.06 (1.05–1.07)**
Pack-years (quartiles) <8.6	352 (14.7)	36 (8.4)	0.53 (0.35–1.18)
8.6 < 14.5	345 (14.4)	32 (7.5)	0.67 (0.42–1.06)
14.5 < 21.75	365 (15.2)	49 (11.5)	1.05 (0.70–1.58)
21.75+	354 (14.7)	175 (41.0)	**4.11 (3.01–5.62)**
**Impact of quitting**			
Reference^*^	986 (65.0)	135 (70.6)	**1.00**
Duration since quitting (tertiles) <15 years	158 (10.4)	22 (11.5)	1.11 (0.69–1.79)
15–27 years	175 (11.5)	22 (11.5)	1.13 (0.73–1.75)
28+ years	199 (13.1)	12 (6.3)	**0.44 (0.26–0.74)**

* Non-smokers.

Adjustments: Year of birth, community size, educational attainment, nasal snuff package-years, years of exposure to hardwood dust, asbestos, organic solvents, use of nasal spray/nasal lavage and ever-use of insecticides in homes.

Analyses by quartiles of pack-years of cigarette smoking showed a peak OR of 4.11 (95% CI 3.01-5.62) in the 4^th^ quartile (exposure to 21.75 pack-years or more). When investigating the impact of quitting cigarette smoking there was an obvious decrease from an OR of 1.11 (95% CI 0.69-1.79) in smokers quitting less than 15 years ago to an OR of 0.44 (95% CI 0.26-0.74) in those who quit 28 or more years ago.

### Exposure to wood dust

We analyzed exposure to softwood dust, chipboard dust, and hardwood dust on risk of nasal cancers. There was no increased risk after exposure to softwood dust or chipboard dust (OR for exposure for at least 1 year: softwood dust 0.51 [95% CI 0.25–1.05]; chipboard dust 0.66 [95% CI 0.25–1.72]). Ever-exposure to hardwood dust resulted in a doubling of risk (OR 2.33, 95% CI 1.40–3.91) with an increase per year of exposure of 6% (95% CI 3–10%) (Table 4). Stratified analyses by cancer site found a significant increase of 6% (95% CI 3–8%) in malignancies of the paranasal sinuses and of 4% in malignancies of the nasal cavity (95% CI 1–7%). In analyses stratified by smoking status, never-smokers had much larger risk increases than smokers, displaying a nearly 5-fold increase for ever-exposure to hardwood dust (OR 4.89, 95% CI 1.92–12.49) and a 6-fold increase in those exposed for 24 years or more (OR 6.09, 95% CI 1.56–23.75). The risks in smokers of exposure to hardwood dust were lower (ever-exposure OR 2.42, 95% CI 1.103–5.68 and exposure for 24+ years OR 4.04, 95% CI 1.2313.2) for 24+ years OR 8.69, 95% CI 1.36–55.42). Analysis by histologic classification showed that increased risks were exclusively for adenocarcinomas (exposed for at least 1 year: OR 18.98, 95% CI 8.24–43.71).

**Table 4:** Association of exposure to hardwood dust with nasal malignancies

	**Controls (%)**	**Cases (%)**	**OR (95%CI)**
**All study participants**			
Reference^*^	2331 (97.1)	393 (92.0)	**1.00**
Exposed for at least 1 year^§^	70 (2.9)	34 (8.0)	**2.33 (1.40–3.91)**
Per year of exposure^§^	70 (2.9)	34 (8.0)	**1.06 (1.03–1.10)**
Exposure (tertile) < 8 years	23 (1.0)	6 (1.4)	2.08 (0.56–7.74)
8 < 24 years	23 (1.0)	9 (2.1)	1.26 (0.51–3.18)
24+ years	24 (1.0)	19 (4.5)	**13.07 (3.44–49.70)**
**Smokers**			
Reference^*^	1370 (96.8)	273 (93.5)	**1.0**
Exposed for at least 1 year^§^	45 (3.2)	19 (6.5)	**2.42 (1.03–5.68)**
Per year of exposure^§^	45 (3.2)	19 (6.5)	**1.07 (1.02–1.12)**
Exposure (tertile) < 8 years	16 (1.1)	4 (1.4)	2.72 (0.56–13.14)
8 < 24 years	16 (1.1)	5 (1.7)	1.65 (0.53–5.18)
24+ years	13 (0.9)	10 (3.4)	**8.69 (1.36–55.42)**
**Never-smokers**			
Reference^*^	961 (97.5)	120 (88.9)	**1.00**
Exposed for at least 1 year^§^	25 (2.5)	15 (11.1)	**6.09 (1.56–23.75)**
Per year of exposure^§^	25 (2.5)	15 (11.1)	**1.09 (1.02–1.16)**
Exposure (tertile) < 8 years	7 (0.7)	2 (1.5)	2.84 (0.24–34.27)
8 < 24 years	7 (0.7)	4 (3.0)	3.20 (0.34–30.07)
24+ years	11 (1.1)	9 (6.7)	**22.25 (2.05–241.02)**

^*^never exposed to hardwood dust.

^§^daily/several days per week.

Adjustments: Year of birth, community size, educational attainment, cigarette pack-years (all study participants), snuff package-years, years of exposure to asbestos, organic solvents, use of nasal spray/nasal lavage and ever-use of insecticides in homes, duration of exposure to softwood dust and to chipboard dust.

### Asbestos

In the total study population, there was only a marginally significant increased risk per year of asbestos exposure in cases with primary malignancy localized in the paranasal sinuses (OR 1.03, 95% CI 1.00–1.06). In smokers, the OR per year of exposure to asbestos was 1.04 (95% CI 1.01–1.06; Table 5). There was an increase across tertiles of exposure duration up to an OR of 2.30 (95% CI 1.17-4.53) in those exposed for 14 years or longer. Significant increases were found in malignancies of the paranasal sinuses and of the nasopharynx (per year of exposure: OR 1.06, 95% CI 1.01–1.13 and OR 1.05, 95% CI 1.01–1.10, respectively). In never-smoking men, no statistically significant increase in risk of exposure to asbestos was found.

**Table 5:** Association of exposure to asbestos with nasal malignancies

	**Controls (%)**	**Cases (%)**	**OR (95% CI)**
**All study participants**			
Reference^*^	2105 (87.7)	360 (84.3)	**1.0**
Ever exposed for at least 1 year^§^	296 (12.3)	67 (15.7)	0.81 (0.47–1.39)
Per year of exposure^§^	296 (12.3)	67 (15.7)	1.02 (1.00–1.04)
Exposure (tertiles) < 5 years	92 (3.8)	11 (2.6)	0.55 (0.24–1.24)
5 < 14 years	106 (4.4)	26 (6.1)	1.13 (0.56–2.28)
14+ years	98 (4.1)	30 (7.0)	1.34 (0.32–5.59)
**Smokers**			
Reference^*^	1237 (87.4)	246 (84.5)	**1.0**
Ever exposed for at least 1 year^§^	178 (12.6)	46 (15.8)	1.23 (0.74–1.91)
Per year of exposure^§^	178 (12.6)	46 (15.8)	**1.03 (1.01–1.06)**
Exposure (tertiles) < 5 years	58 (4.1)	8 (2.7)	0.75 (0.31–1.84)
5 < 14 years	66 (4.7)	14 (4.8)	0.93 (0.48–1.84)
14+ years	54 (3.8)	24 (8.2)	**2.30 (1.17–4.53)**
**Never-smokers**			
Reference^*^	868 (88.0)	114 (84.4)	**1.0**
Ever exposed for at least 1 year^§^	118 (12.0)	21 (15.6)	0.41 (0.08–2.03)
Per year of exposure^§^	118 (12.0)	21 (15.6)	1.17 (0.42–3.28)
Exposure (tertiles) < 5 years	34 (3.4)	3 (2.2)	0.59 (0.15–2.28)
5 < 14 years	40 (4.1)	12 (8.9)	1.17 (0.42–3.28)
14+ years	44 (4.5)	6 (4.4)	0.60 (0.15–2.29)

* never exposed to asbestos.

^§^daily/several days per week.

Adjustments: Year of birth, community size, educational attainment, pack-years (all study participants), snuff package-years, years of exposure to hardwood dust, organic solvents, use of nasal spray/nasal lavage and ever-use of insecticides in residences.

### Organic solvents

Exposure to organic solvents was determined both by questions about exposure during any occupation as well as by a detailed questionnaire module about specific tasks in metal-work. Using these questionnaire moduls we could not detect any correlation with nasal cancer, but the numbers were too low to make firm conclusions. The more general questions, however, indicated an increase in risk for ever-exposure to organic solvents of 56% (95% CI 17–101%), with an increase per year of exposure of 2% (95% CI 1–3%) (Table 6). A similar risk increase applied to malignancies of the nasal cavity and of the paranasal sinuses, but not to nasopharyngeal carcinomas.

**Table 6:** Association of organic solvent exposure with nasal malignancies

	**Controls (%)**	**Cases (%)**	**OR (95% CI)**
**All study participants**			
Reference^*^	1815 (75.6)	281 (65.8)	**1.0**
Ever exposed for at least 1 year^§^	586 (24.4)	146 (34.2)	**1.56 (1.17–2.01)**
Per year of exposure^§^	586 (24.4)	146 (34.2)	**1.02 (1.01–1.03)**
Exposure (tertiles) < 7 years	198 (8.2)	44 (10.3)	**1.57 (1.03–2.38)**
7 < 20 years	183 (7.6)	37 (8.7)	1.23 (0.77–1.96)
20+ years	205 (8.5)	65 (15.2)	**1.75 (1.19–2.58)**
**Smokers**			
Reference^*^	1064 (75.2)	185 (63.4)	**1.00**
Ever exposed for at least 1 year^§^	351 (24.8)	107 (36.6)	**1.95 (1.34–2.83)**
Per year of exposure^§^	351 (24.8)	107 (36.6)	**1.02 (1.01–1.04)**
Exposure (tertiles) < 7 years	123 (8.7)	31 (10.6)	**2.08 (1.19–3.65)**
7 < 20 years	116 (8.2)	25 (8.6)	1.45 (0.76–2.77)
20+ years	112 (7.9)	51 (17.5)	**2.24 (1.31–3.82)**
**Never-smokers**			
Reference^*^	751 (76.2)	96 (71.1)	**1.00**
Ever exposed for at least 1 year^§^	235 (23.8)	39 (28.9)	1.06 (0.61–1.84)
Per year of exposure^§^	235 (23.8)	39 (28.9)	0.99 (0.97–1.02)
Exposure (tertiles) < 7 years	75 (7.6)	13 (9.6)	1.98 (0.84–4.65)
7 < 23 years	80 (8.1)	14 (10.4)	0.61 (0.22–1.67)
23+ years	80 (8.1)	12 (8.9)	0.96 (0.44–2.11)

^*^never exposed to organic solvents.

^§^daily/several days per week.

Adjustments: Year of birth, community size, educational attainment, pack-years (all study participants), snuff package-years, years of exposure to hardwood dust, asbestos, use of nasal spray/nasal lavage and ever-use of insecticides in residences.

The results of an analysis by tertiles of exposure years indicated a possible bimodal distribution of risk, as only the first and third tertiles had significantly increased ORs (1.57 [95% CI 1.03–2.38] and 1.75 [95% CI 1.19–2.58], respectively). Stratified analyses by smoking status indicated an even higher increase in risk in smokers, but no increased risk in never-smokers.

### Other risk factors

Prolonged use of nasal sprays, nasal drops or nasal lavage, i.e., daily use for at least 1 month, was associated with an increased risk of nasal cancer (OR 1.59, 95% CI 1.04–2.44; Table 7). This risk was obvious in the nasopharynx only (ever use: OR 2.2, 95% CI 1.27–4.07; per year of use: OR 1.04, 95% CI 1.01–1.08). A history of diseases of the nose and of the paranasal sinuses was not associated with any increase in risk. Malignancies of any kind in next-of-kin were not associated with nasal cancer.

**Table 7:** Other risk factors associated with nasal malignancies

**Use of nasal lavage/nasal sprays**	**Controls (%)**	**Cases (%)**	**OR (95% CI)**
**All study participants**			
Reference^*^	2.196 (91.5)	381 (89.2)	**1.0**
Ever use daily for at least 1 month	205 (8.5)	46 (10.8)	**1.59 (1.04–2.44)**
Per year of use	205 (8.5)	46 (10.8)	1.01 (.98–1.04)
**Smokers**			
Reference^*^	1.296 (91.6)	265 (91.1)	1.00
Ever use daily for at least 1 month	119 (8.4)	27 (9.3)	1.70 (.92–3.14)
Per year of use	119 (8.4)	27 (9.3)	1.01 (.97–1.05)
**Never-smokers**			
Reference^*^	900 (91.3)	116 (85.9)	1.00
Ever use daily for at least 1 month	86 (8.7)	19 (14.1)	.89 (.37–2.13)
Per year of use	86 (8.7)	19 (14.1)	.98 (.92–1.04)
**Use of insecticides in homes**			
**All study participants**			
Reference^§^	2126 (88.5)	351 (82.2)	**1.00**
Ever use	275 (11.5)	76 (17.8)	**1.48 (1.04–2.11)**
**Smokers**			
Reference^§^	1226 (86.6)	238 (81.5)	1.00
Ever use	189 (13.4)	54 (18.5)	1.17 (0.71–1.91)
**Never-smokers**			
Reference^§^	900 (91.3)	113 (83.7)	1.00
Ever use	86 (8.7)	22 (16.3)	1.78 (.84–3.78)

* never used nasal sprays/nasal lavage daily for 1 month.

^§^ never used domestic insecticides.

Adjustments: nasal lavage/nasal sprays: year of birth, community size, educational attainment, pack-years (total population & smokers), snuff package-years, years of exposure to hardwood dust, asbestos, organic solvents, domestic use of insecticides.

Adjustments: domestic use of insecticides: year of birth, community size, educational attainment, pack-years (total population & smokers), snuff package-years, years of exposure to hardwood dust, asbestos, organic solvents, use of nasal spray/nasal lavage.

Occupational use of herbicides and insecticides, ascertained by detailed survey questionnaire modules for occupation as farmer, gardener or forester, was not associated with any increase in risk of nasal cancer. In contrast to these findings, ever-use of insecticides in private residences was associated with a nearly 50% increase in nasal cancer in the total study population (OR 1.48, 95% CI 1.04–2.11; Table 7), and was mainly due to an increase in malignancies of the nasopharynx (OR 1.80, 95% CI 1.08–3.00). In smokers, the increase in risk of cancer at the nasopharynx was even greater (OR 2.41, 95% CI 1.27–4.77).

## Discussion

### Nasal snuff

We could identify six studies [3-8] that dealt with snuff as a potential risk factor for malignancies of the nasal cavity, paranasal sinuses and nasopharynx, four of which were case-control studies [5-8]. These studies, however, all had some specific limitations. Either the composition of snuff contained several other potentially toxic substances besides tobacco powder (3,7) or “snuff” related both to snuff used nasally as well as orally or exclusively orally (5-8).

Regarding CNC and CPNS, our study was larger than the above-mentioned studies and, in addition, adjustment was achieved for a variety of potential risk factors. When combining all cancer sites and controlling for additional risk factors, we found an increased risk of nasal cancer for use of nasal snuff nearly identical to that reported by Brinton et al. (OR 1.47, 95% CI 0.8–2.8 vs. OR 1.45, 95% CI 0.88–2.38 in our study). The OR increased to 1.71 (95% CI 0.91–3.21) when we restricted the analysis to CPNS. Subgroup analyses in our study suggested that the increased risks of nasal snuff most probably were restricted to users of nasal snuff who were former smokers or who were currently smoking. If there is any increased cancer risk in non-smokers, it is likely to be considerably smaller than in smokers. However, because of the relatively small subgroup of never-smokers in our study (135 cases and 986 controls), the power to detect such a risk might have been too small. Power calculations showed that an OR of 2.0 could be detected with such a sample size. A comparison of package-years of snuff by smoking status shows that never-smoking controls used 19.5 package-years of nasal snuff compared with 17.81 in cases, whereas smoking controls used 14.56 package-years vs. 27.08 in cases. Interestingly, the Expert Committee convened by the International Agency for Research on Cancer, which in 2004 reviewed the available scientific evidence for the carcinogenicity of smokeless tobacco [1, p. 366], came to the conclusion: “Studies on nasal use of snuff did not provide conclusive evidence of a relationship with cancer.”

### Smoking

Smoking as a risk factor for nasal cancer has been investigated by several groups [5-22]. All risk increases reported in these studies, either for ever-smoking or for quartiles of pack-years, were well within the range observed in our study. There were, however, some studies reporting no increase in risk of nasal cancer from smoking [7,23-25]. The impact of quitting smoking has been analyzed in only a few studies [10,13,14], but in none of these publications was the decrease in risk significant. In contrast, our study showed a clear decrease in risk from an OR of 1.11 (95% CI 0.69-1.79) in smokers who quit less than 15 years ago to an OR of 0.44 (95% CI 0.26-0.74) in smokers who quit 28 or more years previously. As the risk was considerably smaller than that for non-smokers, it is possible that men who quit smoking and abstained from smoking for a longer period not only changed from smoking to non-smoking but may also have improved other aspects of their lifestyle (nutrition, physical activity).

### Hardwood dust

Exposure to hardwood dust is a well established risk factor for the development of adenocarcinoma of the nasal cavities [25,26]. Although Blot and co-authors [27], in a review of North American studies, found marginally significant increases in risk, probably as a result of very few exposed cases, other studies showed significantly increased risks or much larger risk ratios [9,28-34], and increasing risks with increasing dose or time of exposure [30-32]. Our study showing an OR of 2.33 for exposure of at least 1 year (95% CI 1.40–3.91) as well as an increase over tertiles of exposure years from 1.04 to 5.34 (95% CI 2.55–11.20) is well in agreement with the latter studies. When stratifying our analyses by smoking status we found larger risks in never-smokers than in smokers. This suggests a lack of interaction between smoking and exposure to hardwood dust on the risk of nasal cancer. Increases in risk were restricted to carcinoma of the nasal cavity and of the paranasal sinuses. We could also confirm no increase in risk with exposure to softwood dust, as others have shown repeatedly. We found increased risks for adenocarcinomas only, as reported by other researchers. It is interesting that in none of the recent case–control studies [31,33] wood dust has been analyzed by its origin (hardwood, softwood, chipboard).

### Organic solvents

Exposure to organic solvents in our study emerged as a consistent risk factor for carcinoma of the nasal cavities. An analysis by tertiles of exposure years indicated a bimodal distribution, as only the first and third tertiles showed significantly increased ORs (1.57, 95% CI 1.03–2.38 and 1.75, 95% CI 1.19–2.58, respectively). A similar finding was reported by Hildesheim and co-authors [31] in a case–control study on carcinoma of the nasopharynx. For an exposure time of less than 10 years, they reported an OR of 1.5 (95% CI 0.99–2.3) and no risk increase with longer exposure periods (OR 0.93, 95% CI 0.61–1.4).

### Asbestos

Exposure to asbestos was a strong risk factor for carcinoma of the paranasal sinuses and of the nasopharynx in ever-smokers, but not in never-smokers. Maximum risk was found in the third tertile of exposure years for malignancies of the paranasal sinuses (OR 5.37, 95% CI 1.18–24.36). The increase in risk per year of exposure in our study was 6% (95% CI 0–13%) for malignancies of the paranasal sinuses and 5% (95% CI 1–10%) for malignancies of the nasopharynx. In a pooled analysis of 12 case–control studies on sinonasal cancers, Luce and co-authors [35] found inconsistent results, as men with the lowest probability of exposure to asbestos fibers showed the largest ORs. However, the authors had not included smoking as a potential confounder nor did they stratify by smoking status. To emulate the analysis conducted by Luce and co-authors, we dropped pack-years of cigarettes smoked from our full model on paranasal sinus carcinoma and found slightly increased risk for tertiles of exposure (in the 3^rd^ tertile OR 2.46, 95% CI 1.11–5.45 vs. 2.36, 95% CI 1.00–5.57, with inclusion of pack-years as a confounder). It is our opinion that this finding should lead to a cautious interpretation of confounding in the case of strong risk factors, e.g., smoking.

### Use of insecticides in homes

An increased risk associated with use of insecticides in homes, as found in our study, has not been described in any of the published studies. Brinton and co-authors [5] found an OR of 1.41 for the combined application of insecticides, pesticides, and herbicides in men. Zou and co-authors [36] reported a non-significant increase in risk of NPC after use of pesticides (OR 1.6, 95% CI 0.9–2.8), whereas Zhu and co-authors [20] found a strong impact of pesticide use on sinonasal malignancies (OR 5.9, 95% CI 1.5–23.7). A recent investigation of Tisch and co-workers [37] offered a plausible pathophysiologic explanation for our findings, as a variety of insecticides (pentachlorophenol, lindane, transfluthrin, cyfluthrin and natural pyrethrum) showed strong genotoxic effects when applied to isolated mucosal cells from inferior and middle nasal conchae. The increase in risk was restricted to never-smoking men. This is in contrast to findings for other risk factors where risk increases in smokers were larger than in never-smokers. However, when analyzing the prevalence of never-smoking and of use of insecticides in homes by educational attainment, both characteristics were greatest in best-educated men.

### Analysis of potential biases

Biased estimates for exposures in case–control studies may arise from differing responses of cases and controls in interviews or likewise differing responses of cases or controls and of next-of-kin. Next-of-kin interviews were conducted in 134 of 427 cases (31%) and in 19 of 2401 controls (0.8%).

The results of an analysis of responses of cases and of their proxies (Table 8) demonstrated that, except for exposure to hardwood dust, there was no significant difference in any of the major risk factors identified in this case–control study. Assuming that there is under-reporting of hardwood dust exposure by next-of-kin, this would lead to an underestimate of the actual OR.

**Table 8:** Analysis of potential bias of 134 next-of-kin interviews in 427 cases

	**Prevalence (%) in interviews conducted with:**		**Chi**^**2**^	**p**
	**Case**	**Next-of-kin**		
**Educational attainment**				
Primary school or less	66.6	70.2	0.61	0.74
Secondary school	18.4	17.2		
College	15.0	12.7		
**Use of tobacco products**				
Smoking (ever)	66.9	71.6	0.96	0.33
Nasal snuff (ever)	6.25	6.72	0.03	0.85
**Exposures**				
Exposure to hardwood dust	12.9	0	18.9	<0.0001
Exposure to asbestos (ever)	15.4	16.4	0.078	0.78
Exposure to solvents (ever)	35.5	31.3	0.70	0.40
Residential use of insecticides	17.8	17.9	0.002	0.97
Nasal lavage/nasal sprays	11.3	12.7	0.16	0.69

A biased estimate of exposures comparing cases and controls could be expected if there was common knowledge of the effect of a potential risk factor for the disease under investigation. Such knowledge could induce cases to attribute their disease to the risk factor, whereas controls would respond indifferently.

Regarding the relevant risk factors investigated in this case–control study, there was no widely available information linking these risk factors to nasal carcinomas. Moreover, questions regarding these risk factors were embedded in a multitude of other questions. None of these questions hinted at a connection with disease.

There was a markedly different distribution of educational attainment in cases vs. controls (Table 1). If this reflected a selective difference between controls and cases, it could lead to biased risk estimates. There are, however, two arguments to refute such an assumption. First, a lower response rate in cases than in controls would lead to a higher proportion of respondents with better educational attainment. This argument is based on findings reached during the conduct of the first German National Health Examination Survey (1984-1985), where interim analyses conducted at increasing levels of response rates showed increasing proportions of participants with lower educational attainment, below average income, without jobs, being smokers or overweight.^a^

Second, most of the established carcinogens tend to be linked either to occupations that require less educational attainment or to lifestyle habits more often found in less well-educated people. Thus, it has to be expected that the educational attainment of cases would reflect exposures and individual risk factors that are more prominent in these social groups. In consequence, more cases would be expected in men with less education. An analysis, stratified by educational attainment (Table 9), confirmed that ORs for tobacco use as well as for exposure to asbestos, hardwood dust and organic solvents were greatest in the least educated men.

**Table 9:** Odds ratios of risk factors stratified by educational attainment

**Risk factor**	**Educational attainment**		
	**Elementary school**	**Secondary school**	**College**
Smoking*	**1.066 (1.053–1.80)**	**1.050 (1.026–1.075)**	1.030 (0.994–1.068)
Use of nasal snuff*	1.008 (1.0–1.018)	0.84 (0.17–4.08)	0.985 (0.948–1.023)
Exposure to asbestos*	**1.023 (1.002–1.044)**	1.017 (0.959–1.078)	0.867 (0.648–1.161)
Exposure to hardwood dust*	**1.040 (1.021–1.059)**	1.004 (0.875–1.153)	0.955 (0.738–1.235)
Exposure to solvents*	**1.017 (1.005–1.029)**	1.00 (0.962–1.04)	1.042 (0.994–1.068)
Domestic use of insecticides (ever)	1.21 (0.77–1.90)	1.61 (0.66–3.91)	**3.86 (1.53–9.70)**
Use of nasal lavage/nasal sprays*	1.003 (0.968–1.039)	1.052 (0.990–1.117)	0.968 (0.882–1.062)

* Per 1 year of exposure to asbestos, hardwood dust, organic solvents, per 1 pack-year of cigarettes, 1 package-year of nasal snuff, 1 month of use of nasal lavage/spray for 1 year.

Adjustments for age, community size and for all other risk factors (duration of exposure or pack-years/package-years respectively, if appropriate).

### Strengths and limitations of the study

A major limitation of our study is the relatively small response rate in cases, i.e., 55% vs. 62% in controls. Usually one would expect higher response rates in cases, but as the recruitment of cases in our study was carried out by staff of tumor registries or of ENT departments, not by members of our study group, no attempts to encourage participation could be undertaken. With an increase in response rate an increased proportion of persons exposed to specific risk factors could be expected, and a lower response rate in cases could lead to smaller risk estimates.

Another shortcoming of our study was the retrospective recruitment of cases. As all malignancies included in our study are rather rare, prospective recruitment would most certainly have resulted in fewer deceased cases than encountered with the present retrospective design, but the study would not have been feasible owing to an extremely long prospective recruitment period required.

Our study is the first study to deal with the impact of nasal snuff on nasal cancer in an industrialized country. Thus the results regarding nasal snuff need confirmation by future studies.

There are several strengths of our study. It was a relatively large population-based case–control study. Moreover, the possibility of stratifying the analyses by smoking status indicated that smoking, besides being a risk factor itself, could act as a potent modifier for other risk factors.

## Conclusions

The results from our study confirm the role of smoking and exposure to hardwood dust and asbestos as carcinogens for nasal carcinoma. In addition, we found increased risks associated with exposure to organic solvents. Use of insecticides in homes is suggested as a potential new risk factor. Exposure to nasal snuff or asbestos dust leads to an increased risk, mostly in past and current smokers. In nearly all the analyses, increases in risk were more prominent in smokers than in non-smokers.

### Ethical Review Committees

The study design has been approved by the Ethical Review Committees of the Bremen State Chamber of Physicians, of the Bavarian State Chamber of Physicians, of the Baden-Württemberg State Chamber of Physicians, of the Augsburg Central Hospital, and of the Tübingen University Hospital.

### Endnotes

^a^The first author of this paper was chairman of the German Cardiovascular Prevention Study. The National Health Examination Surveys provided reference data for community-based preventions, conducted in 6 different communities throughout Germany.

## Abbreviations

CI: Confidence interval; CNC: Cancers of the nasal cavity; CPNS: Cancers of the paranasal sinuses; ENT: Ear, nose and throat; NPC: Nasopharyngeal cancer; OR: Odds ratio. One packyear, referring to cigarette smoking, is equivalent of smoking of one pack of cigarettes per day for one year. One package-year, referring to use of nasal snuff, is equivalent for the use of one package of nasal snuff per week for one year.

## Competing interest

The study was financed in full by Pöschl Tabak GmbH & Co KG, Geisenhausen, Germany. The contract for the conduct of the study with E.G. was signed after Dr. Pöschl, CEO of Pöschl Tabak GmbH & Co KG, had agreed to the following terms: 1. no interference with design, conduct, analysis, publication of study results; 2. establishment of an independent scientific advisory board; 3. conduct of a pilot study. Throughout the entire study these terms have been strictly adhered to. The authors declare no conflict of interest.

## Authors’ contribution

EMG designed the study, developed the study protocol, provided all of the statistical analyses, wrote the first and the final version of the manuscript. KHG revised the first and subsequent versions of the manuscript. WA organized and oversaw the conduct of interviews, revised the first and subsequent versions of the manuscript. RH, RL, BS and HPZ contributed patients as cases for the study and revised the manuscript. HM contributed to the final version of the study protocol, re-classified histo-pathologic findings of all cases and contributed to revisions of the manuscript. All authors read and approved the final manuscript.

## Authors’ information

EMG, MD, PhD, is Professor emeritus for medical statistics and epidemiology, Faculty of Health Sciences, Bremen University, Bremen Germany. He is currently member of the Department of Health Economics, Health Policy and Health Care Research, Center for Health Policy Research, Bremen University, and CEO of Epi. Consult GmbH, Musweiler, Germany. KHG, MD, MPH, is senior research fellow at the Department of Cancer Epidemiology, German Cancer Research Centre, Heidelberg, Germany. WA, PhD, is Director of the Department of Epidemiologic Methods and Etiologic Research, BIPS Institute for Epidemiology and Prevention Research, and Deputy Director of BIPS, Bremen University, Bremen, Germany.

RH, MD, PhD, is director of the Department of Otorhinolaryngology, University of Wuerzburg, Wuerzburg, Germany. RL, MD, PhD, is director of the Department of Otorhinolaryngology – Head and Neck Surgery, Albert-Ludwigs University Medical School, Freiburg, Germany. HM, MD, PhD, is director of the Department of Otorhinolaryngology, Head and Neck Surgery, Bundeswehr Hospital, Ulm, Germany. Bernhard Schick, MD, PhD, is director of the Department of Otorhinolaryngology, Saarland University, Homburg/Saar, Germany. HPZ, MD, PhD, is director of the Department of Otolaryngology, University of Tübingen, Tübingen, Germany.

## Contributing ENT hospitals and tumour registries (key persons)

Tumour registries Tumorregister München des Tumorzentrums München (Prof. Dr. Dieter Hölzel), München, Germany, Tumorzentrum Regensburg – Epidemiologisches Krebsregister (Prof. Dr. Ferdinand, Hofstädter, Dr. Sabrina Klinkhammer-Schalke), Regensburg, Germany, Tumorzentrum Erlangen – Bevölkerungsbezogene Krebsregistrierung für, Mittelfranken (Prof. Dr. Heinrich Iro, Dr. Sabrina Petsch), Erlangen, Germany.

## ENT hospitals

Klinikum der Universität München - Klinik und Poliklinik für Hals-, Nasen und, Ohrenkrankheiten (Prof. Dr. med. Alexander Berghaus), München, Germany, Technische Universität München - Klinikum rechts der Isar – Hals-, Nasen-Ohrenklinik und Poliklinik (Prof. Dr. W. Arnold), München, Germany, Zentralklinikum Augsburg – HNO-Klinik (Prof. Dr. Franz Xaver Brunner), Ausgburg, Germany, Universitätsklinikum Würzburg – Klinik und Poliklinik für Hals-, Nasen- und, Ohrenkranke (Prof. Dr. Jan Helms), Würzburg, Germany, Katharinenhospital Stuttgart – Hals-Nasen- Ohrenklinik (Prof. Dr. Rudolf Hagen), Stuttgart, Germany, Klinikum der Universität Regensburg – HNO-Klinik (Prof. Dr. Jürgen Strutz), Regensburg, Germany, Universitätsklinikum Erlangen - Hals-Nasen-Ohrenklinik – Kopf- und Halschirurgie, (Prof. Dr. Heinrich Iro), Erlangen, Germany, Klinikum Nürnberg – Hals-Nasen-Ohrenklinik (Prof. Dr. Jürgen Theissing, Prof. Dr. Viktor Bonkowsky), Universitätsklinikum Heidelberg – Hals-Nasen-Ohrenheilkunde, (Prof. Dr. Peter K. Plinkert, PD Dr. A. Dietz), Heidelberg, Germany, Universitätsklinikum Freiburg – Universitätsklinik für Hals-, Nasen- und, Ohrenheilkunde (Prof. Dr. Dr.h.c. Roland Laszig), Freiburg, Germany, Universitätsklinikum Freiburg – Zahn-, Mund- und Kieferheilkunde – Klinik und, Poliklinik für Mund-, Kiefer- und Gesichtschirurgie (Prof. Dr. Dr. Rainer, Schmelzeisen), Freiburg, GermanyvUniversitätsklinikum Ulm – Hals- Nasen- und Ohrenheilkunde (Prof. Dr. Gerhard, Rettinger), Ulm, Germany, Universitätsklinikum Tübingen – Universitätsklinik für Hals,- Nasen- und, Ohrenheilkunde (Prof. Dr. Dr.h.c.mult. Hans Peter Zenner), Tübingen, Germany, Klinikum im Haus Gesundbrunnen Heilbronn – Klinik für Hals-Nasen-Ohrenkrankheiten und plastische Chirurgie (Prof. Dr. Claus Naumann), Heilbronn, Germany.

## Scientific Advisory Board

Prof. Dr. Jenny Chang-Claude (Chair), German Cancer Research Center, Department of Clinical Epidemiology, Heidelberg, Germany, Prof. Dr. Karl-Heinz Jöckel, Institute for Medical Informatics, Biostatistics and Epidemiology, Essen University Hospital, Essen, Germany, Prof. Dr. Heinz Maier, ENT Department, Armed Forces Hospital, Ulm, Germany, Prof. Dr. Uwe John, Institute for Epidemiology and Social Medicine, Ernst-Ludwig-Arndt University, Greifswald, Germany.

## Pre-publication history

The pre-publication history for this paper can be accessed here:

http://www.biomedcentral.com/1471-2407/12/506/prepub
